# Editorial: Avian microbiome: from embryonic development to adulthood

**DOI:** 10.3389/fphys.2023.1211911

**Published:** 2023-05-03

**Authors:** Monika Proszkowiec-Weglarz, Brian Oakley, Laura E. Ellestad, Sundus Javed

**Affiliations:** ^1^ Animal Biosciences and Biotechnology Laboratory, Agriculture Research Service, United States Department of Agriculture, Beltsville, MD, United States; ^2^ College of Veterinary Medicine, Western University of Health Sciences, Pomona, CA, United States; ^3^ Department of Poultry Science, University of Georgia, Athens, GA, United States; ^4^ Department of Biosciences, COMSAT, University of Islamabad, Islamabad, Pakistan

**Keywords:** gastrointestinal tract, microbiota, fungi, development, metatranscriptome, metabolome, domestic birds

Avian gastrointestinal and reproductive microbiota are composed of bacteria, fungi, viruses, and protists and characterized by commensal, symbiotic, and pathogenic relationships with the host. Microbial populations play an important role in modulating host growth and reproductive performance, including nutrient digestion, absorption, and utilization, metabolic and reproductive efficiency, pathogen exclusion, endocrine activity, and immune system development. In chickens, symbiotic relationships between the host and the microbiota have been characterized by nutrient exchange, modulation of the immune system, pathogen exclusion, and gastrointestinal tract (GIT) and reproductive physiology. Microbiota composition and function can be affected by many factors, including age, host genotype and sex, diet composition and form, feed additives such as antibiotics, probiotics, prebiotics, postbiotics, synbiotics, phytobiotics and bacteriophages, stress, and location in the GIT or reproductive tract.

Most microbiome research in avian species has been focused on the GIT of domestic poultry such as broilers, laying hens, and, to some extent, turkeys. The reproductive microbiota in domestic poultry, as well as both the intestinal and reproductive microbiota of wild birds, remain largely unknown. Moreover, most current microbiome research primarily focuses on compositional studies using 16S ribosomal RNA (rRNA), and sequencing and functional studies remain elusive. The goal of this Research Topic was to provide a comprehensive overview of the avian microbiome that includes studies addressing intestinal microbiomes in both domestic and wild birds, including compositional and functional studies.

Gastrointestinal microbiota composition varies by niche, and therefore studies characterizing avian GIT microbiome can be greatly affected by the experimental design choices and specific methodologies used. Weinroth et al. focused on standardizing microbiota an analysis protocol, specifically 16S rRNA sequencing, to examine common sampling practices in broiler chicken studies. Microbiota collected from the GIT was compared to those from cloacal swabs, and it was concluded that cloacal swabs are not a good approximation of the actual internal community at other GIT locations. They also found that sample sizes over 7.6 birds increase new observed amplicon sequence variants by less than 1%, and that cecal pair mates provide adequate replication.

Microbiota may be modulated by many factors during development, as well as in mature birds. Roth et al. characterize the microbiota in GIT from the crop to the ceca in two different laying hens breeds that were fed different level of Ca- and P-supplemented diets. These minerals play a pivotal role in many physiological processes in birds, including homeostatic mechanisms involved in Ca and P utilization for eggshell deposition and bone remodeling in highly productive laying hens. They have found that supplementation of Ca and P at 20% below the recommendation level had only a minor effect on microbiota composition in the GIT in comparison to the effects of genetic background of the birds. Van Syoc et al. showed that metformin, a drug commonly used off-label for polycystic ovary syndrome that benefits metabolic and reproductive health, has the potential to modulate the microbiota of GIT in broiler breeder hens. Besides the modulatory effect on microbiota, metformin was shown to have beneficial effects on metabolism and reproduction in breeder hens by decreasing body weight and increasing egg production.

Bacterial composition of competitive exclusion products (CEP) and their efficacy in reducing *Salmonella* in poultry have been studied by Lee D. et al. They revealed that bacterial community composition of master stock or seeds, as well as CEP commercial lots, were not a good predictor of their potency in reducing *Salmonella* abundance. In a second paper, Lee et al. characterize the pioneer colonizers of the chicken GIT. They have shown that CEP administered at hatch positively effects intestinal morphology, including villi height, goblet cell production, and feed efficiency. Moreover, administration of CEP stabilized the ileal microbiota diversity and promoted *Clostridium* abundance. They also showed that *Bacteroides* may act as pioneer colonizers in chicks, facilitating successional colonization of anaerobic bacteria. Administration of defined CEP formulations have similar effects on ileal morphology but lower the abundance of *Lactobacillus*.

The dynamic changes in microbiota composition in ceca and litter from chicken raised in two different houses from hatch to pre-harvest were determined by Zwirzitz et al. The animal (ceca) and environmental (litter) bacterial communities underwent consistent changes over time and the changes were correlated with the differences in environmental factors such as humidity, temperature, and ammonia level between the houses. The Shterzer et al. paper focused on differences in GIT bacterial communities between modern and slow growing broiler breeder lines. Selection for growth and high meat yield resulted in changes to GIT microbiota, probably due two physiological changes of the intestinal mucosal layer. As the authors concluded, it is still unclear if the changes in microbiota are part of the mechanism affecting the growth or are secondary results of other physiological changes accelerating the growth.

Bacteria are the major component of microbiota, and their role in host health and growth has been extensively studied and is beginning to be understood; however, the role of the fungal population in the chicken GIT during microbiota development is not as well characterized. Temporal changes in the chicken mycobiota during the first 2 weeks post-hatch and due to delayed access to feed early post-hatch have been investigated by Davies et al. The authors show transient changes in mycobiota during post-hatch development in the GIT and determined that negative effects of delayed access to feed early post-hatch are not likely related to the changes in developmental pattern of the fungal population in the GIT.

The review by Jadhav et al. addresses the connection between microbes, short chain fatty acids (SCFA), serotonergic system, and behavior in avian species. The authors speculated that considering the nature of SCFA interactions and the conserved molecular and behavioral features of the serotonergic system, the chicken may be an emergent translational model for identifying underlying mechanisms of change within the GIT-microbiome-brain axis.

